# XhetRel: a pipeline for X heterozygosity and relatedness analysis of sequencing data

**DOI:** 10.1093/bioadv/vbag002

**Published:** 2026-01-22

**Authors:** Barış Salman, Nerses Bebek, Sibel Uğur İşeri

**Affiliations:** Department of Genetics, Aziz Sancar Institute of Experimental Medicine, Istanbul University, Istanbul, 34093, Turkey; Graduate School of Health Sciences, Istanbul University, Istanbul, 34126, Turkey; Izmir Biomedicine and Genome Center, Izmir, 35340, Turkey; Department of Neurology, Istanbul Faculty of Medicine, Istanbul University, Istanbul, 34093, Turkey; Department of Genetics, Aziz Sancar Institute of Experimental Medicine, Istanbul University, Istanbul, 34093, Turkey

## Abstract

**Motivation:**

Verification of sample sex is an essential quality control step in next-generation sequencing studies, typically assessed from genomic data. Clustering individuals by X chromosome heterozygosity (Xhet) and incorporating relatedness estimates offers a practical first-pass screen for potential sex label errors, sample mix-ups, and pedigree inconsistencies. To better interpret Xhet based patterns, we further investigated the biological and technical origins using the 1000 Genomes Project dataset.

**Results:**

We developed XhetRel, a user-friendly workflow and notebook application that computes Xhet and performs relatedness estimation directly from VCF files. As a fully genotype-based approach, XhetRel enables both sex-based clustering and relatedness assessment as an initial quality control (QC) step in NGS. XhetRel serves groups without bioinformatics infrastructure, users requiring a browser-based QC tool, and workflow developers seeking a modular Nextflow component. Our investigation into the sources of Xhet variation highlighted important limitations in sequencing and variant-calling approaches. In particular, specific pseudogenes and gene clusters, such as SLC25A5 and the GAGE cluster, as recurrent contributors to misleading variant allele fractions.

**Availability and implementation:**

The source code and data are available at Figshare (doi: 10.6084/m9.figshare.28280414). XhetRel can be executed locally via Nextflow or accessed directly through the online Collab notebook at https://colab.research.google.com/drive/1ep69JvXLwK5ndHUQ8qIGTWvauzsTW9fi.

## 1 Introduction

With the increasing feasibility of exome and genome sequencing (ES, GS), research on the contribution of rare variants to both rare and complex diseases has gained substantial momentum (Hiz Kurul *et al.* 2022, Epi25 Collaborative 2024). This advancement has led to large-scale collection, processing, and storage of biological samples to ensure generation of reliable genomic data. In particular, trio-based next-generation sequencing (NGS) studies involving affected children and their parents have become a cornerstone in the investigation of pediatric neurodevelopmental disorders (Wright *et al.* 2018, Chengyan *et al.* 2024). However, errors may arise at several stages of the workflow, including inaccurate self-reporting, data entry or labeling mistakes, sample mix-ups, cross-contamination, and analytical artifacts arising from sequencing or variant-calling processes. In addition, unrecognized sex chromosome aneuploidies can further confound downstream analyses and lead to misleading results, highlighting the importance of implementing robust, data-driven quality control procedures.

X chromosome heterozygosity (Xhet) analysis has been applied for sample sex verification in large datasets as part of quality control, and also in a single case for assessing X chromosomal status in somatic mosaicism in conjunction with karyotype analysis (Do *et al.* 2015, Taudien *et al.* 2016, Akçakaya *et al.* 2019). In addition, relatedness analysis is used to infer the degree of genetic identity between individuals, serving as an essential check to confirm reported familial relationships, such as parent-offspring pairs in trio studies. When combined, these two approaches allow early flagging of sex or pedigree related inconsistencies early in the analysis workflow, providing an opportunity for researchers to verify, and reassess samples using alternative methods if needed.

From an evolutionary perspective, X and Y chromosomes originated from a shared ancestral autosomal pair (Lahn and Page 1999). Over time, they have accumulated repetitive and highly homologous regions, which complicate sequence alignment and variant calling (Pinto *et al.* 2023). Despite continuous methodical improvements, sex chromosomes are still often excluded from bioinformatic analyses due to their repetitive content, sequence homology, and complex genomic architecture, as well as biological variability, arising from mosaicism and aneuploidies (Webster *et al.* 2019). Notably, the Y chromosome was the last human chromosome to be completely sequenced, reflecting the persistent technical challenges posed by its complex structure (Rhie *et al*, 2023).

In sequencing datasets, heterozygous variants on the X chromosome may be observed in XY individuals; however, these do not reflect true biological variation, since XY individuals carry only a single copy of X chromosome and are hemizygous for both sex chromosomes. The exception occurs in the pseudoautosomal regions (PARs) 1 and 2, which together span ∼3 Mb and are located at the distal ends of the X and Y chromosomes. These regions exhibit high sequence homology that enables pairing and recombination between two chromosomes (reviewed in Wang *et al.* 2022). Because both X and Y contribute to alleles in the PARs, XY individuals may therefore show both homozygous and heterozygous genotypes in PARs, even though the rest of the X chromosome is expected to be hemizygous.

The presence of heterozygous variants on the X chromosome outside the PARs in XY individuals represents a technical artifact rather than a biological phenomenon. In this context, quantifying Xhet provides an interpretable metric for sex-based clustering as part of sample quality assessment.

Here, we present XhetRel, a user-friendly notebook and workflow that computes Xhet and relatedness metrics directly from variant call format (VCF) files for early sample-level quality control. Although X-het-based metrics have previously been applied in a limited number of studies, an accessible and easy-to-use implementation that performs these computations directly from VCF files has not been available. Moreover, combining Xhet-based clustering with relatedness assessment across multiple samples in a single lightweight tool provides a practical capability for combined sample-level QC. In addition, XhetRel implements a parametric Xhet calculation, allowing users to define alternative filtering conditions such as allele fraction, depth, or genotype quality according to their analytical needs. Because XhetRel operates entirely on genotype data, it enables both sex-based clustering and relatedness assessment without requiring alignment data. We further investigated the genomic sources of Xhet by analyzing chromosome X variants from the 1000 Genomes Project (1KGP) (Auton *et al.* 2015).

## 2 Methods

XhetRel was implemented as a Nextflow pipeline (Di Tommaso *et al.* 2017) and tested using two benchmark trios annotated as mother, father and son in the Genome in a Bottle (GIAB) dataset, representing Ashkenazi Jewish (AJ) and Han Chinese (C) ancestry, respectively, based on VCF files generated from Illumina paired-end genome sequencing (Zook *et al.* 2016). Exact data links are provided in Supplementary material, available as supplementary data at *Bioinformatics Advances* online.

**Figure 1:**
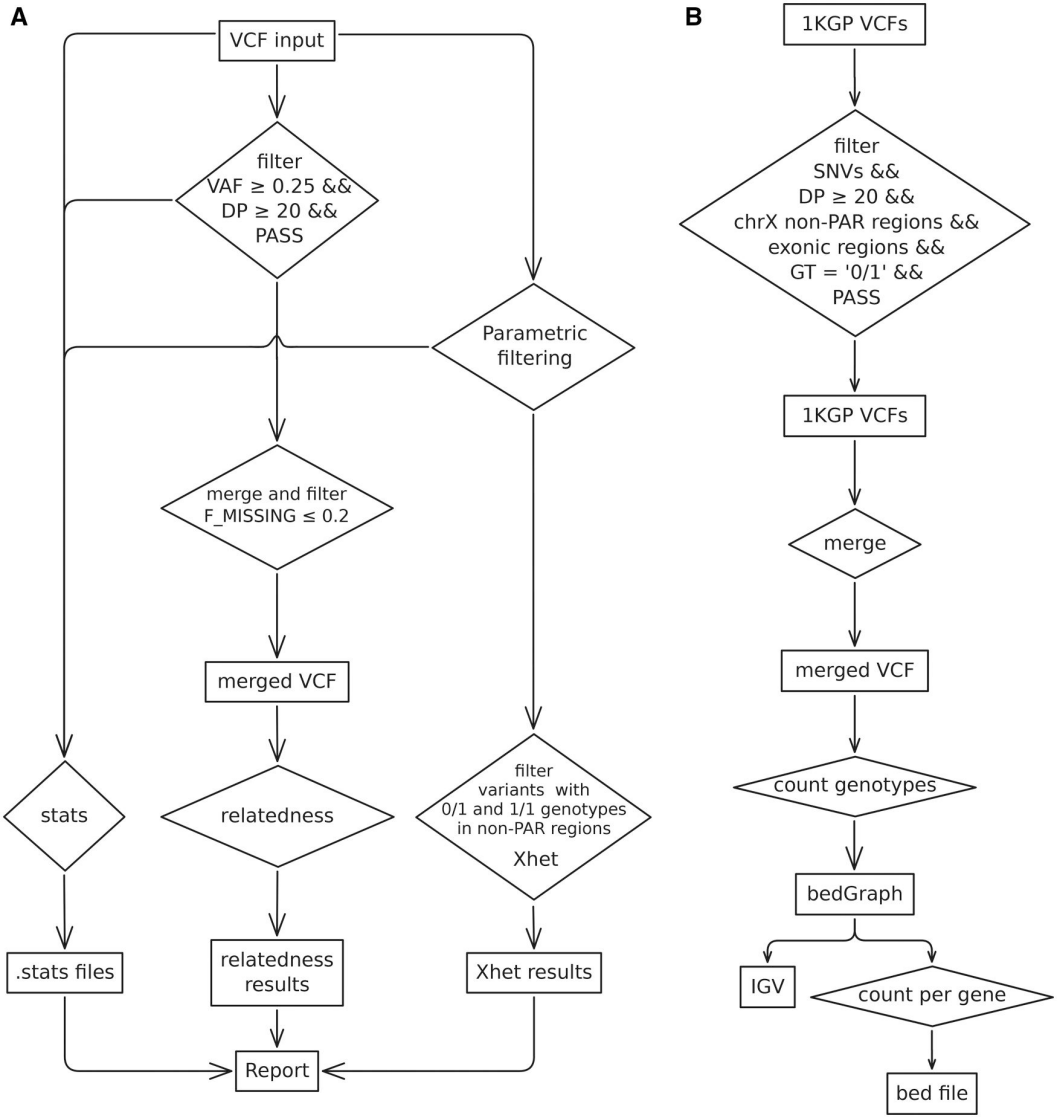
Overview of the analysis workflows diagrams. (A) XhetRel pipeline for sex inference and relatedness estimation from VCF files. (B) Workflow diagram used for identifying Xhet in the 1000 Genomes Project dataset.

For relatedness analysis, individual VCF files are first filtered for variants with allele fractions >0.25 and read depth >20, then merged into a single file. Variants with >20% missing genotypes are further excluded (Fig. 1A). The robust relationship inference method implemented in VCFtools was applied for relatedness (Manichaikul *et al.* 2010, Danecek *et al.* 2011).

For X chromosome analysis, non-PAR variants are extracted from the chrX:2 781 479–153 925 834 interval, the smallest common region excluding PAR1 and PAR2 shared among the GRCh37/hg19, GRCh38/hg38, and T2T assemblies using BCFtools, which is also used to retain only variants with genotypes of 0/1 and 1/1 (Danecek *et al.* 2021). Xhet is calculated as the proportion of heterozygous variants relative to the total number of variants on chromosome X using AWK. Results are visualized via a custom plot generated in MultiQC (Ewels *et al.* 2016).

Further validation was performed using an in-house ES dataset, where Xhet was recalculated with XhetRel using different quality parameters. We additionally assessed the accuracy of sex inference by comparing XhetRel results with the DRAGEN v4.2 ploidy estimator. As an independent control, matching sex estimations from SNP array data were used as labels for the logistic regression model.

To explore the sources of heterozygosity, we analyzed X chromosome genotypes from 1223 samples labeled as “male” in the 1000 Genomes Project data (https://www.internationalgenome.org/data-portal/sample), restricting the analysis to exonic single nucleotide variants (SNVs) and applying three variant allele-fraction (VAF) thresholds (25 ≤ VAF ≤ 75, 33 ≤ VAF ≤ 66, and 45 ≤ VAF ≤ 55). The number of samples with heterozygous calls each region was counted, and the top 20 regions were annotated (Fig. 1B).

Paralogous gene annotations were retrieved from the Comparative Genomics section of Ensembl database (https://www.ensembl.org/info/genome/compara/index.html), while pseudogene lists for each gene were retrieved from the HUGO Gene Nomenclature Committee (HGNC) database (https://genenames.org). Pseudogene alignments were verified using UCSC BLAT (https://genome.ucsc.edu/cgi-bin/hgBlat).

## 3 Results

### 3.1 Performance of XhetRel on benchmark trios and validation

**Figure 2:**
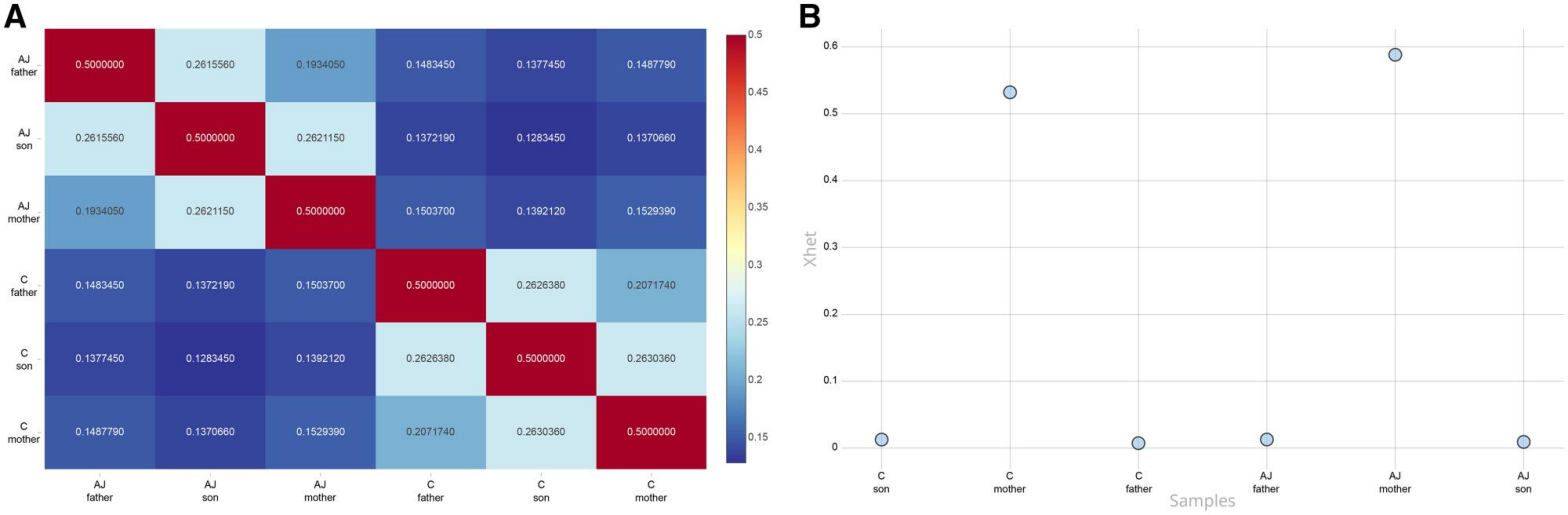
Relatedness and Xhet plots generated by MultiQC. (A) Heatmap of pairwise relatedness values. (B) Custom plot illustrating Xhet for the same individuals. AJ: Ashkenazi Jewish trio samples; C: Han Chinese trio samples.

XhetRel was applied to two benchmark trios from the Genome in a Bottle Consortium. The pairwise kinship coefficients (ϕ) computed using VCFtools showed the expected relationship structure (Fig. 2A). Parent-offspring pairs in both trios demonstrated coefficients of ∼0.26, consistent with first-degree relatedness, whereas the parents showed lower values (0.19–0.20), reflecting their unrelated status. As expected, comparisons between individuals from different trios yielded kinship values approaching zero (0.12–0.15).

Xhet values derived from non-PAR regions of chromosome X clearly separated male and female samples (Fig. 2B). Maternal samples showed Xhet values around 0.5, whereas paternal and offspring samples were close to zero, consistent with the hemizygous state of the X chromosome in males. These patterns demonstrate that VCF-derived XhetRel output simultaneously reveals expected sex-associated clustering and reflects familial relationships, without requiring external metadata.

**Figure 3:**
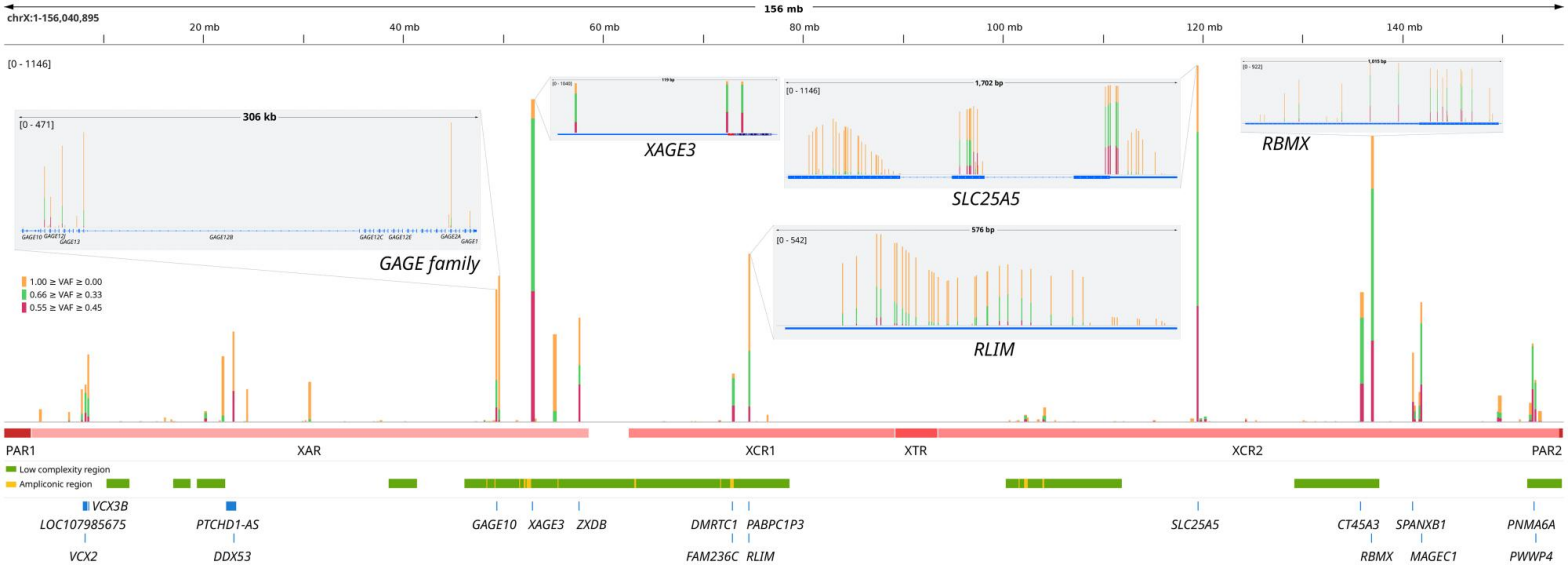
Genotype counts across chromosome X for alternative allele fraction thresholds. Regions of low complexity, ampliconic loci, and major X chromosome segments (PAR, XAR, XCR, XTR) are highlighted. PAR, pseudoautosomal region; XAR, X-added region; XCR, X-conserved region; XTR, X-transposed region.

We next assessed XhetRel in an independent cohort of 143 ES samples matched with SNP array data and DRAGEN v4.2 ploidy calls. Xhet based sex clustering was in complete concordance with both DRAGEN’s chromosomal ploidy and SNP array sex assignments. As expected, median X chromosome coverage was lower in the Xhet-defined male cluster, whereas the female cluster showed near-zero coverage on chromosome Y, consistent with biological ploidy (Fig. 1A and B, available as supplementary data at *Bioinformatics Advances* online). To assess robustness, Xhet was recalculated under alternative filtering conditions (allele fraction, depth, genotype quality). Although absolute Xhet values shifted across parameter settings, the sex-based separation remained robust (Fig. 1C, available as supplementary data at *Bioinformatics Advances* online). A single deviation was observed in a female sample with extended runs of X chromosome homozygosity likely due to parental consanguinity (Fig. 2, available as supplementary data at *Bioinformatics Advances* online), which reduced Xhet but remained outside the male cluster. Logistic regression analysis further confirmed that Xhet alone is sufficient to genotypically separate male and female samples, with complete cluster separation (Fig. 3 and Table 1, available as supplementary data at *Bioinformatics Advances* online). The standard Xhet filtering scheme was also applied to 25 male and 25 female samples from the 1000 Genomes Project, again yielding a clean bi-modal separation (Fig. 4, available as supplementary data at *Bioinformatics Advances* online).

**Table 1:** Genotype counts for the top 20 X-linked genes contributing to Xhet in male samples under different thresholds.^a^

25 ≤ VAF ≤ 75		33 ≤ VAF ≤ 66		45 ≤ VAF ≤ 55	
Gene symbol	Count	Gene symbol	Count	Gene symbol	Count
*SLC25A5*	30 694	*SLC25A5*	10 454	*SLC25A5*	3799
*RLIM*	10 839	*RBMX*	5552	*RBMX*	1858
*PABPC1P3*	10 824	*XAGE3*	3155	*XAGE3*	1207
*RBMX*	10 370	*PABPC1P3*	2456	*MAGEC1*	444
*XAGE3*	3537	*RLIM*	2456	*PABPC1P3*	301
*MAGEC1*	2120	*MAGEC1*	1415	*RLIM*	301
*ZXDB*	1135	*CT45A3*	718	*CT45A3*	248
*CT45A3*	937	*ZXDB*	480	*ZXDB*	187
*GAGE2A*	606	*PWWP4*	329	*PWWP4*	144
*PTCHD1-AS*	597	*PNMA6A*	249	*DDX53*	128
*DDX53*	596	*DMRTC1*	228	*PTCHD1-AS*	128
*SPANXB1*	572	*FAM236C*	228	*PNMA6A*	111
*GAGE12B*	564	*DDX53*	216	*DMRTC1*	101
*PWWP4*	517	*PTCHD1-AS*	216	*FAM236C*	101
*SPANXD*	493	*VCX3B*	149	*SPANXB1*	89
*VCX3B*	436	*SPANXD*	144	*GAGE12J*	52
*GAGE13*	393	*GAGE10*	140	*VCX3B*	48
*VCX*	338	*SPANXB1*	131	*GAGE10*	39
*GAGE10*	310	*LOC107985675*	107	*LOC107985675*	36
*LRCH2*	290	*VCX2*	107	*VCX2*	36

a The total number of unique heterozygous genotype calls are 71 553, 27 504, and 9243 for the VAF cut-offs 25 ≤ VAF ≤ 75, 33 ≤ VAF ≤ 66, and 45 ≤ VAF ≤ 55, respectively. Variants mapping to overlapping gene pairs (PABPC1P3, RLIM and DDX53, PTCHD1-AS) are listed twice in the table.

### 3.2 Distribution of X-linked heterozygosity in 1000 genomes males

To investigate the origin of X heterozygosity in male samples, we quantified heterozygous calls from 1223 males in the 1000 Genomes Project using three VAF thresholds (25%–75%, 33%–66%, 45%–55%). Across these thresholds, we identified a total of 71 553, 27 504, and 9243 heterozygous positions, respectively. The highest recurrence was observed in four major evolutionary regions of chromosome X (Webster *et al.* 2019): X-added region (XAR; recently added X region in the eutherian lineage), X-conserved region (XCR; evolutionary conserved X region between therian mammals), X-transposed region (XTR; duplicated region from X to the Y after human-chimpanzee divergence), and XCR2, with densities ranging from 2.3 to 789 heterozygous calls per Mb. Ampliconic regions 5 and 9 showed the greatest concentration of heterozygosity, with densities of 6945.5 and 1013.2 per Mb, respectively (Cotter *et al.* 2016). We visualized the distribution of heterozygous sites across chromosome X and annotated them according to the major evolutionary regions (XAR, XTR, XCR) as well as low-complexity and ampliconic segments (Fig. 3). Genomic intervals for major evolutionary and minor ampliconic regions obtained from Webster *et al.* (2019) and Cotter *et al.* (2016), respectively, were remapped to hg38 using UCSC liftOver.

### 3.3 Gene-level contributors to recurrent male Xhet

Table 1 lists the top 20 genes contributing to recurrent heterozygous calls across male samples. The *SLC25A5* gene ranked first under all filtering thresholds, with nearly 3800 heterozygous calls even at the most stringent VAF cutoff (45 ≤ VAF ≤ 55). UCSC-BLAT hg38 alignment of the *SLC25A5* transcript (1,307 bases: NM_001152.5/ENST00000317881.9) revealed strong homology to 10 autosomal loci (>80% identity over >1 kb), nine of which correspond to processed pseudogenes (*SLC25A5P1-9*). These pseudogenes are classified as transcribed processed or processed-only pseudogenes, indicating that multiple retrotransposition events occurred during genome evolution. (Table 2). In addition, *SLC24A4* on chromosome 4 and *SLC25A6* on chromosomes X and Y are paralogous genes for *SLC25A5*. These findings highlight the extensive homology of *SLC25A5* across multiple chromosomes, posing significant challenges for short-read NGS approaches and resulting in ambiguous outcomes.

**Table 2:** UCSC-BLAT alignment results for the SLC25A5 mRNA sequence against the hg38 reference genome.^a^

Query	Start	End	Identity	Strand	Chr	Start (bp)	End (bp)	Span size (bp)	Gene included
NM_001152.5	1	1307	100.00%	F	chrX	119 468 444	119 471 396	2953	*SLC25A5*
NM_001152.5	1	1227	93.90%	F	chr7	324 714 29	32 472 998	1570	*SLC25A5P5*
NM_001152.5	5	1234	93.90%	F	chr7	54 419 371	54 420 600	1230	*SLC25A5P3*
NM_001152.5	6	1222	92.90%	R	chr22	42 000 445	42 002 033	1589	*SLC25A5P1*
NM_001152.5	14	1223	92.90%	R	chr2	33 839 529	33 840 742	1214	*SLC25A5P2*
NM_001152.5	2	1203	92.30%	F	chr6	121 653 739	121 654 924	1186	*SLC25A5P7*
NM_001152.5	1	1230	92.00%	R	chr9	32 333 195	32 334 431	1237	*SLC25A5P8*
NM_001152.5	14	1230	91.30%	R	chr4	186 328 455	186 329 663	1209	*SLC25A5P6*
NM_001152.5	48	1179	88.80%	F	chr13	57 314 711	57 316 417	1707	*SLC25A5P4*
NM_001152.5	100	347	87.70%	F	chr4	185 143 400	185 144 927	1528	*SLC25A4*
NM_001152.5	58	1224	85.10%	R	chr5	75 752 294	75 753 498	1205	*SLC25A5P9*
NM_001152.5	91	810	81.20%	R	chrY	1 387 280	1 391 991	4712	*SLC25A6*
NM_001152.5	91	810	81.20%	R	chrX	1 387 280	1 391 991	4712	*SLC25A6*

a The top row corresponds to the primary query match on chromosome X. Nine additional hits are detected on autosomal loci involving pseudogenes. One alignment is on SLC254A4 on chromosome 4, and two hits on sex chromosomes (excluding the query locus).

Similar patterns were observed for *RBMX*, which has 36 paralogs and 5 pseudogenes (*RBMXP1-5*). Among these, the paralog *RBMXL1*, located on chromosome 1, shows the highest similarity to the X-linked *RBMX* gene, with 99% identity in the cDNA level and 95% identity across the full gene sequence.

A comparable signature is seen in the primate-specific *GAGE* gene family, which is located as a 16-copy cluster at Xp11.23 with high sequence identity among members. The *GAGE* locus represents a relatively recent evolutionary expansion in primates (Liu *et al.* 2008). All *GAGE* copies contain an L1 element, except for *GAGE1*, which is considered to be the ancestral gene (Gjerstorff and Ditzel 2008).

Together, these findings demonstrate that male X heterozygosity in short-read sequencing arises not from true diploidy but from mis-alignment to duplicated or paralogous regions, reinforcing the need for chromosome-aware QC approaches.

## 4 Discussion

### 4.1 XhetRel as a lightweight QC workflow

We have developed XhetRel as an accessible and modular workflow that can be executed either directly in a Google Colab notebook with no local installation requirements or integrated into larger analysis pipelines through Nextflow and Docker. The workflow enables early detection of major sample-level errors, including sex mismatches and pedigree inconsistencies, prior to downstream variant analysis. Unlike tools that estimate sex from alignment files (e.g. DRAGEN, XYalign, seGMM) (Webster *et al.* 2019, Liu *et al.* 2022), XhetRel computes Xhet parametrically from VCF files generated later in variant analysis pipelines, making it particularly useful when alignment files are unavailable.

Although the workflow is designed for ease of use, larger datasets may benefit from running locally rather than in Colab, for improved performance and scalability.

It is important to note that pairwise relatedness estimates obtained through the *relatedness2* package cannot be used as evidence for ACMG *de novo* classification (PS2), as the method does not perform Mendelian consistency checks. Moreover, because XhetRel combines Xhet with kinship coefficients, it cannot distinguish between same-sex first-degree relationships, such as mother-daughter pairs or father-son pairs, since both the degree of relatedness and the inferred sex category are identical in these cases. As a result, a mother-daughter pair is indistinguishable from one another in the output, and the same applies to a father-son pair.

### 4.2 Insights from 1000 genomes project analyses

Our analysis of male samples from the 1000 Genomes Project demonstrated that apparent heterozygous calls on the X chromosome mainly originate from paralogous and pseudogene regions, particularly involving *SLC25A5*, *RBMX*, and the *GAGE* gene cluster.

Because the human genome contains extensive repetitive content, large and highly duplicated regions often remain unresolved by current sequencing and genome assembly technologies, particularly those based on short-read data (Hoyt *et al.* 2022, Liao *et al.* 2023). These artefactual variant calls are more readily detectable on the hemizygous X chromosome, where any deviation from hemizygosity can be attributed almost exclusively to sequencing or mapping errors. The same types of errors are expected to occur on autosomes as well, but they are more difficult to recognize there because true biological heterozygosity masks them.

Recent complete assemblies of the human genome, including the T2T reference and long-read sequencing technologies, now provide a more reliable framework for resolving highly repetitive and duplicated regions (Miga *et al.* 2020). In future studies, variant allele fraction (VAF) profiles on the X chromosome can be used to recalibrate heterozygosity estimates in short-read datasets and filter out pseudoheterozygous calls that originate from mis-mapped reads in paralogous or pseudogene-rich regions in other regions of the genome.

Taken together, our results demonstrate that XhetRel provides an efficient first-pass QC strategy for sex inference and relatedness screening in sequencing-based studies. However, Xhet values in particular remain sensitive to both technical (variant caller, filtering thresholds, joint vs. single-sample calling) and biological (runs of homozygosity, aneuploidies, structural variation, etc.) sources of variation; therefore, they should be interpreted with caution, preferably within datasets processed through the same pipeline, and confirmed by orthogonal validation whenever inconsistencies are suspected.

## Supplementary Material

vbag002_Supplementary_Data

## Data Availability

The data underlying this article are available in *XhetRel* at https://github.com/barslmn/XhetRel/, and can be accessed with 10.6084/m9.figshare.28280414. Google Colab notebook can be accessed at https://colab.research.google.com/drive/1ep69JvXLwK5ndHUQ8qIGTWvauzsTW9fi.
