# Supplementary material for: XhetRel: a pipeline for X heterozygosity and relatedness analysis of sequencing data
Source: Bioinform Adv. 2026 Jan 22;6(1):vbag002. doi: 10.1093/bioadv/vbag002 (PMC12883445; doi:10.1093/bioadv/vbag002)
Supplement: vbag002_Supplementary_Data [file vbag002_supplementary_data.docx]

**XhetRel: A Pipeline for X Heterozygosity and Relatedness Analysis of** **Sequencing Data Supplementary**

## Comparison of XhetRel with DRAGEN-based ploidy estimation and SNP array sex assignment

Our dataset of 143 exome sequencing (ES) and matching SNP array data had been generated as part of the international Epi25 study (<http://epi-25.org/>). Sequencing produced an average of 62.3 ± 10.9 million aligned reads per sample with a mean coverage depth of 48.27x (standard deviation ± 6.96x). The XhetRel analysis was performed on the joint-called GATK ES-VCF files.

Additional alignment and variant calling from CRAM files were carried out using the Illumina DRAGEN pipeline. X and Y chromosome median coverages are acquired from DRAGEN. SNP array data was processed in GenomeStudio, and only samples with call rates > 0.99 were selected.

Sex assignments derived from DRAGEN ploidy calls, SNP array data, and XhetRel based Xhet calculations (VAF ≥ 0.25, depth ≥ 20, FILTER = PASS) were all concordant. Median X-chromosome coverage was lower in the Xhet-defined male cluster, whereas the female cluster showed near-zero coverage on chromosome Y, consistent with biological ploidy (Supplementary Figure 1A, 1B).

Xhet was recalculated under alternative filtering conditions (allele fraction, depth, genotype quality). Although absolute Xhet values shifted across parameter settings, the sex-based separation remained concordant (Supplementary Figure 1C).


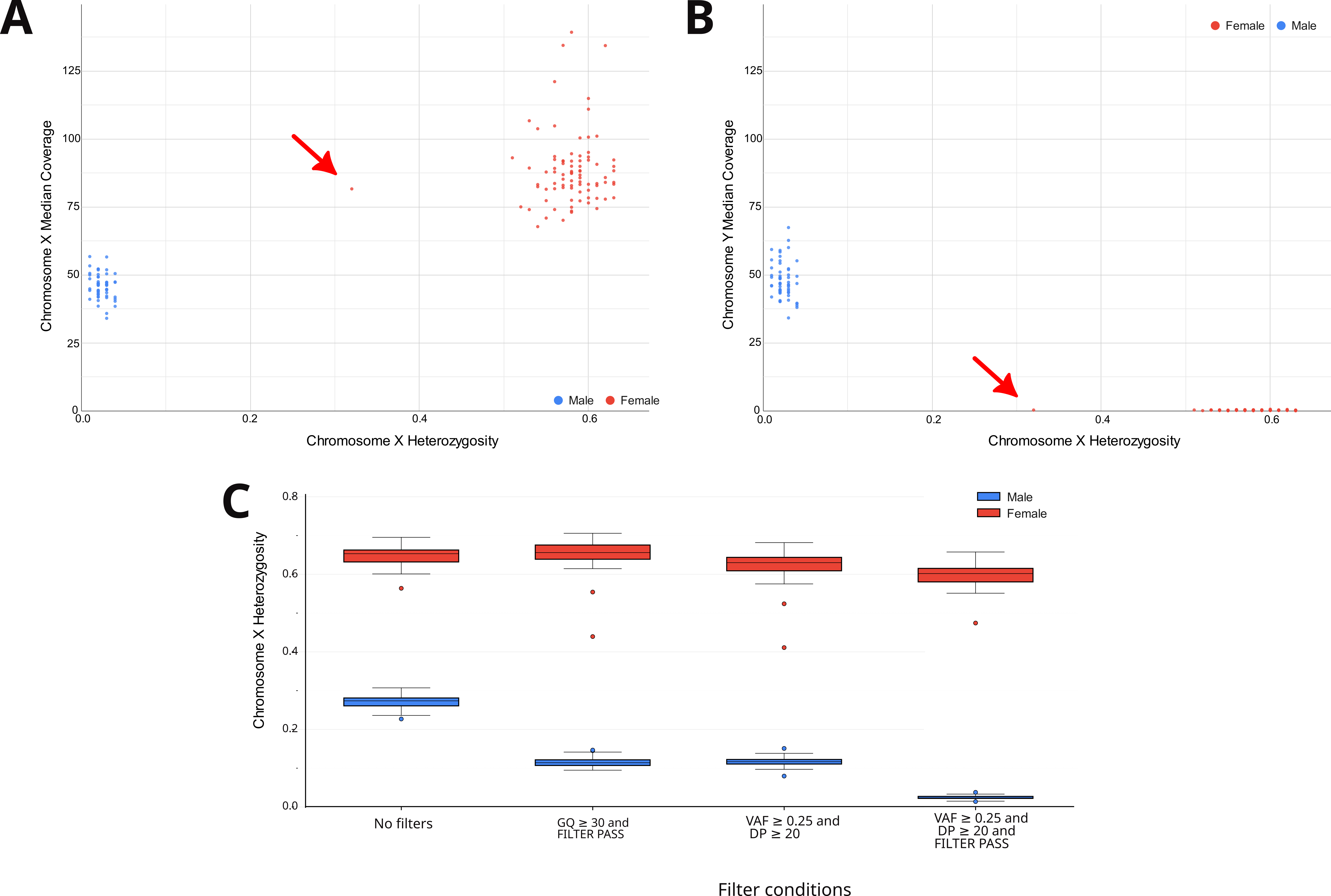
Supplementary Figure 1: A-B. X heterozygosity compared to X and Y median coverages from Dragen. C. X heterozygosity in males and females under different variant filtering conditions: no filters, GQ ≥ 30, and VAF ≥ 0.25 + DP ≥ 20 + FILTER PASS.

## Runs of X-chromosome homozygosity in a female sample

One female sample highlighted with the red arrow in Supplementary Figure 1A and B, despite having a high median X-chromosome coverage (81.67x), showed a noticeably reduced Xhet value (0.32) compared with the rest of the females in the cohort. Inspection of the corresponding SNP array data revealed multiple long runs of homozygosity on chromosome X (hg38; chrX:1,844,162-32,324,821, chrX:73,345,638-78,689,825 and chrX:117,037,257-156,030,895), along with similar extended ROH segments on autosomes, consistent with parental consanguinity as the likely cause of reduced Xhet.


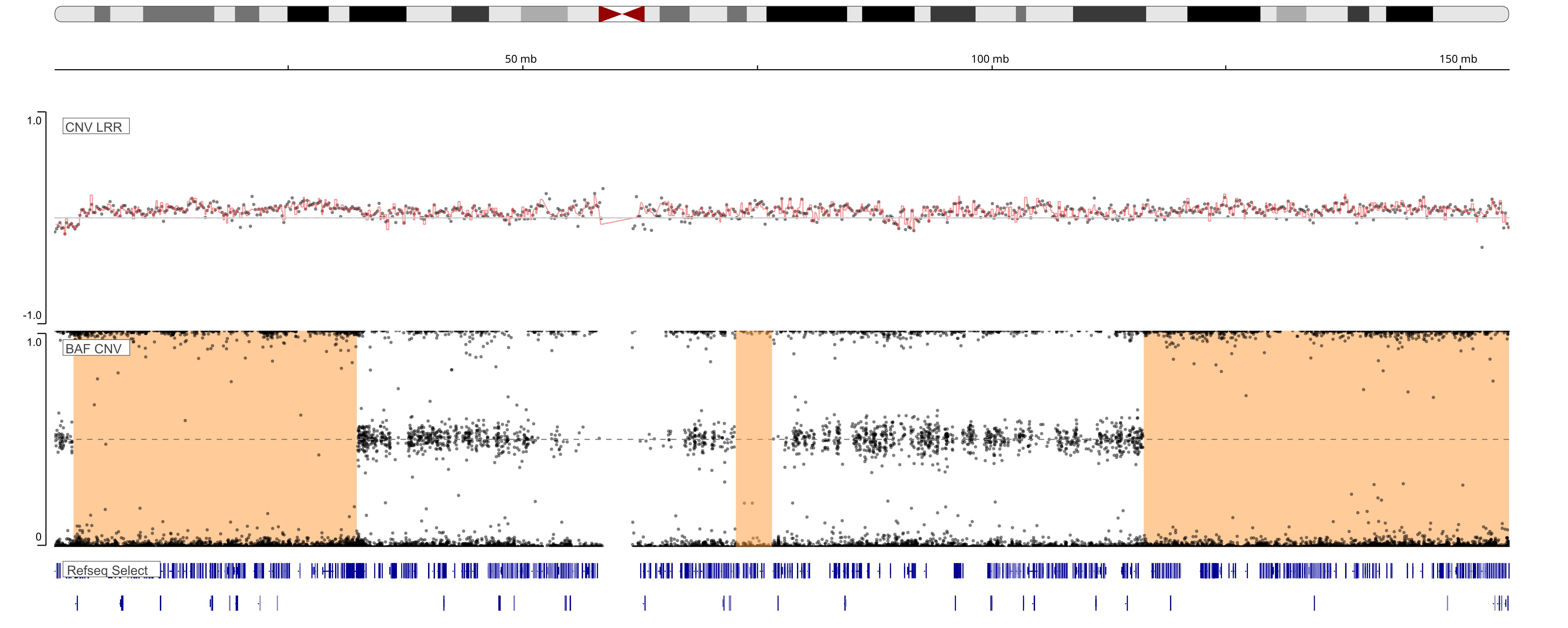
Supplementary Figure 2: Female sample with low X heterozygosity. Long stretches of homozygous regions on chromosome X are detected in SNP array (hg19). Orange regions indicate copy neural loss of heterozygosity.

## X heterozygosity sex estimation

To evaluate the robustness of the X heterozygosity on sex-based clustering, we trained and evaluated a logistic regression model under varying conditions of data quality (read depth and allele fraction) and data completeness. Genetic variants from the ES X chromosome were analyzed, and used SNP array-derived sex estimations as labels. For each sample, we calculated the heterozygous ratio, defined as the number of heterozygous variants divided by the sum of heterozygous and homozygous alternate variants. To assess the impact of data quality, the analysis was stratified into six groups based on read depth (DP) and Variant Allele Fraction (VAF). Three DP ranges were used: 0-10, 10-20, and ≥20. For each DP range, variants were processed either with and without a VAF filter of ≥ 0.25. In all conditions GATK filters were applied and only PASS variants were included.

A logistic regression model was then trained using the heterozygous ratio as the single predictive feature. To evaluate robustness to data sparsity, the analysis was repeated on random subsets of each sample's variants, using 100%, 75%, 50%, 25%, 10%, 5%, and 1% of available variants. Model performance under each quality condition and variant subset was quantified using accuracy and the Area Under the Receiver Operating Characteristic Curve (AUC-ROC). Sex specific threshold for male and female samples were calculated as one standard deviation from the mean, with 95% confidence intervals (CI). The estimation threshold has been calculated as the average of the means.


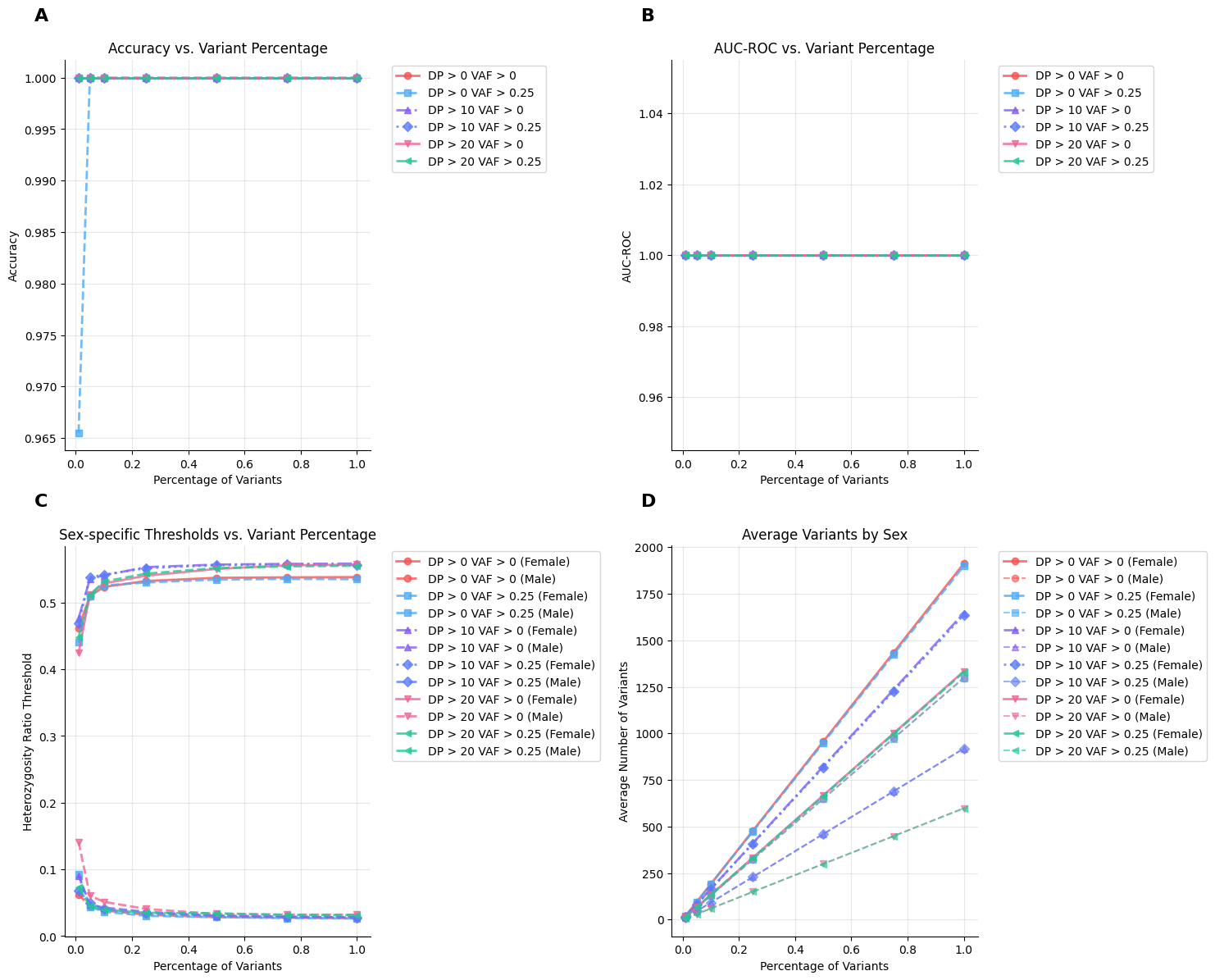
Supplementary Figure 3: These calculations has been performed excluding sample 82. A) Accuracy vs variant percentage over DP and VAF brackets. B) AUC-ROC vs Variant Percentage over DP and VAF brackets. C) Optimal threshold for different variant percentage for different DP and VAF brackets. D) Avarage number of variants over different variant percentages and DP, VAF brackets for males and females.

Supplementary Table 1: These calculations has been performed after excluding the female sample that displayed an unusually low Xhet value (Supplementary Figure 1). The table lists the number of variants used for logistic regression and the resulting optimal thresholds for each subsampling percentage, and for each combination of VAF and DP brackets. CI were calculated at a 0.95 confidence level.

| **Percentage of variants** | **VAF** | **DP** | **Number of variants** | **Female mean** | **Male mean** | **Accuracy** | **AUC ROC** | **Estimation threshold** | **Female threshold CI** | **Male threshold CI** |
| --- | --- | --- | --- | --- | --- | --- | --- | --- | --- | --- |
| 1 | 0 | 0 | 18 | 0.57 | 0.02 | 1 | 1 | 0.26 | 0.440, 0.484 | 0.053, 0.073 |
|  |  | 10 | 15 | 0.6 | 0.03 | 1 | 1 | 0.28 | 0.449, 0.504 | 0.076, 0.107 |
|  |  | 20 | 11 | 0.59 | 0.04 | 1 | 1 | 0.28 | 0.390, 0.459 | 0.119, 0.170 |
|  | 0.25 | 0 | 18 | 0.56 | 0.03 | 0.97 | 1 | 0.28 | 0.414, 0.465 | 0.079, 0.109 |
|  |  | 10 | 15 | 0.58 | 0.02 | 1 | 1 | 0.28 | 0.444, 0.493 | 0.057, 0.081 |
|  |  | 20 | 11 | 0.58 | 0.02 | 1 | 1 | 0.28 | 0.421, 0.477 | 0.059, 0.088 |
| 5 | 0 | 0 | 94 | 0.56 | 0.03 | 1 | 1 | 0.28 | 0.499, 0.521 | 0.040, 0.050 |
|  |  | 10 | 76 | 0.59 | 0.02 | 1 | 1 | 0.27 | 0.524, 0.547 | 0.041, 0.053 |
|  |  | 20 | 58 | 0.59 | 0.03 | 1 | 1 | 0.28 | 0.497, 0.528 | 0.053, 0.069 |
|  | 0.25 | 0 | 94 | 0.56 | 0.02 | 1 | 1 | 0.28 | 0.499, 0.520 | 0.039, 0.049 |
|  |  | 10 | 76 | 0.59 | 0.02 | 1 | 1 | 0.28 | 0.527, 0.549 | 0.043, 0.056 |
|  |  | 20 | 58 | 0.58 | 0.02 | 1 | 1 | 0.28 | 0.498, 0.525 | 0.039, 0.051 |
| 10 | 0 | 0 | 189 | 0.57 | 0.03 | 1 | 1 | 0.28 | 0.515, 0.532 | 0.035, 0.042 |
|  |  | 10 | 153 | 0.59 | 0.03 | 1 | 1 | 0.28 | 0.531, 0.551 | 0.038, 0.047 |
|  |  | 20 | 117 | 0.58 | 0.03 | 1 | 1 | 0.28 | 0.517, 0.541 | 0.045, 0.058 |
|  | 0.25 | 0 | 188 | 0.56 | 0.02 | 1 | 1 | 0.29 | 0.518, 0.534 | 0.033, 0.040 |
|  |  | 10 | 153 | 0.59 | 0.02 | 1 | 1 | 0.29 | 0.533, 0.551 | 0.036, 0.045 |
|  |  | 20 | 117 | 0.58 | 0.02 | 1 | 1 | 0.29 | 0.522, 0.542 | 0.035, 0.044 |
| 25 | 0 | 0 | 474 | 0.56 | 0.02 | 1 | 1 | 0.29 | 0.526, 0.539 | 0.030, 0.034 |
|  |  | 10 | 384 | 0.59 | 0.03 | 1 | 1 | 0.29 | 0.546, 0.561 | 0.034, 0.039 |
|  |  | 20 | 293 | 0.58 | 0.03 | 1 | 1 | 0.29 | 0.532, 0.548 | 0.037, 0.044 |
|  | 0.25 | 0 | 472 | 0.56 | 0.02 | 1 | 1 | 0.27 | 0.524, 0.537 | 0.029, 0.032 |
|  |  | 10 | 382 | 0.59 | 0.02 | 1 | 1 | 0.29 | 0.545, 0.559 | 0.032, 0.037 |
|  |  | 20 | 292 | 0.58 | 0.02 | 1 | 1 | 0.29 | 0.536, 0.551 | 0.032, 0.038 |
| 50 | 0 | 0 | 949 | 0.56 | 0.02 | 1 | 1 | 0.29 | 0.532, 0.543 | 0.027, 0.030 |
|  |  | 10 | 769 | 0.59 | 0.02 | 1 | 1 | 0.29 | 0.550, 0.564 | 0.029, 0.032 |
|  |  | 20 | 587 | 0.58 | 0.02 | 1 | 1 | 0.29 | 0.544, 0.557 | 0.031, 0.036 |
|  | 0.25 | 0 | 944 | 0.56 | 0.02 | 1 | 1 | 0.29 | 0.529, 0.540 | 0.027, 0.030 |
|  |  | 10 | 765 | 0.59 | 0.02 | 1 | 1 | 0.28 | 0.550, 0.562 | 0.029, 0.033 |
|  |  | 20 | 585 | 0.58 | 0.02 | 1 | 1 | 0.29 | 0.545, 0.559 | 0.031, 0.037 |
| 75 | 0 | 0 | 1423 | 0.56 | 0.02 | 1 | 1 | 0.29 | 0.532, 0.543 | 0.026, 0.029 |
|  |  | 10 | 1153 | 0.59 | 0.02 | 1 | 1 | 0.29 | 0.552, 0.564 | 0.028, 0.030 |
|  |  | 20 | 881 | 0.58 | 0.02 | 1 | 1 | 0.29 | 0.550, 0.562 | 0.030, 0.034 |
|  | 0.25 | 0 | 1416 | 0.56 | 0.02 | 1 | 1 | 0.29 | 0.530, 0.541 | 0.026, 0.028 |
|  |  | 10 | 1148 | 0.58 | 0.02 | 1 | 1 | 0.29 | 0.552, 0.563 | 0.027, 0.031 |
|  |  | 20 | 878 | 0.58 | 0.02 | 1 | 1 | 0.26 | 0.548, 0.560 | 0.030, 0.034 |
| 100 | 0 | 0 | 1898 | 0.56 | 0.02 | 1 | 1 | 0.28 | 0.533, 0.544 | 0.026, 0.028 |
|  |  | 10 | 1538 | 0.59 | 0.02 | 1 | 1 | 0.29 | 0.553, 0.564 | 0.027, 0.030 |
|  |  | 20 | 1175 | 0.58 | 0.02 | 1 | 1 | 0.29 | 0.551, 0.563 | 0.030, 0.034 |
|  | 0.25 | 0 | 1888 | 0.56 | 0.02 | 1 | 1 | 0.29 | 0.530, 0.541 | 0.025, 0.027 |
|  |  | 10 | 1531 | 0.58 | 0.02 | 1 | 1 | 0.29 | 0.550, 0.562 | 0.027, 0.029 |
|  |  | 20 | 1171 | 0.58 | 0.02 | 1 | 1 | 0.29 | 0.550, 0.561 | 0.029, 0.034 |

## Xhet analysis from 1000 Genomes project data

25 male and 25 female samples from 1000 genomes project has been analyzed for Xhet using XhetRel with the parameters VAF ≥ 0.25, depth ≥ 20, FILTER = PASS. Data has been acquired from <https://ftp.1000genomes.ebi.ac.uk/vol1/ftp/data_collections/1000G_2504_high_coverage/working/20190425_NYGC_GATK/raw_calls_old/>.

Across all samples, Xhet values clustered consistently with reported sex: female samples formed the higher Xhet group, and male samples formed the lower Xhet group. One female sample, while not clustering within the female group, nevertheless exhibited a lower Xhet value than the majority of female samples, consistent with the pattern observed in Supplementary Figure 1.


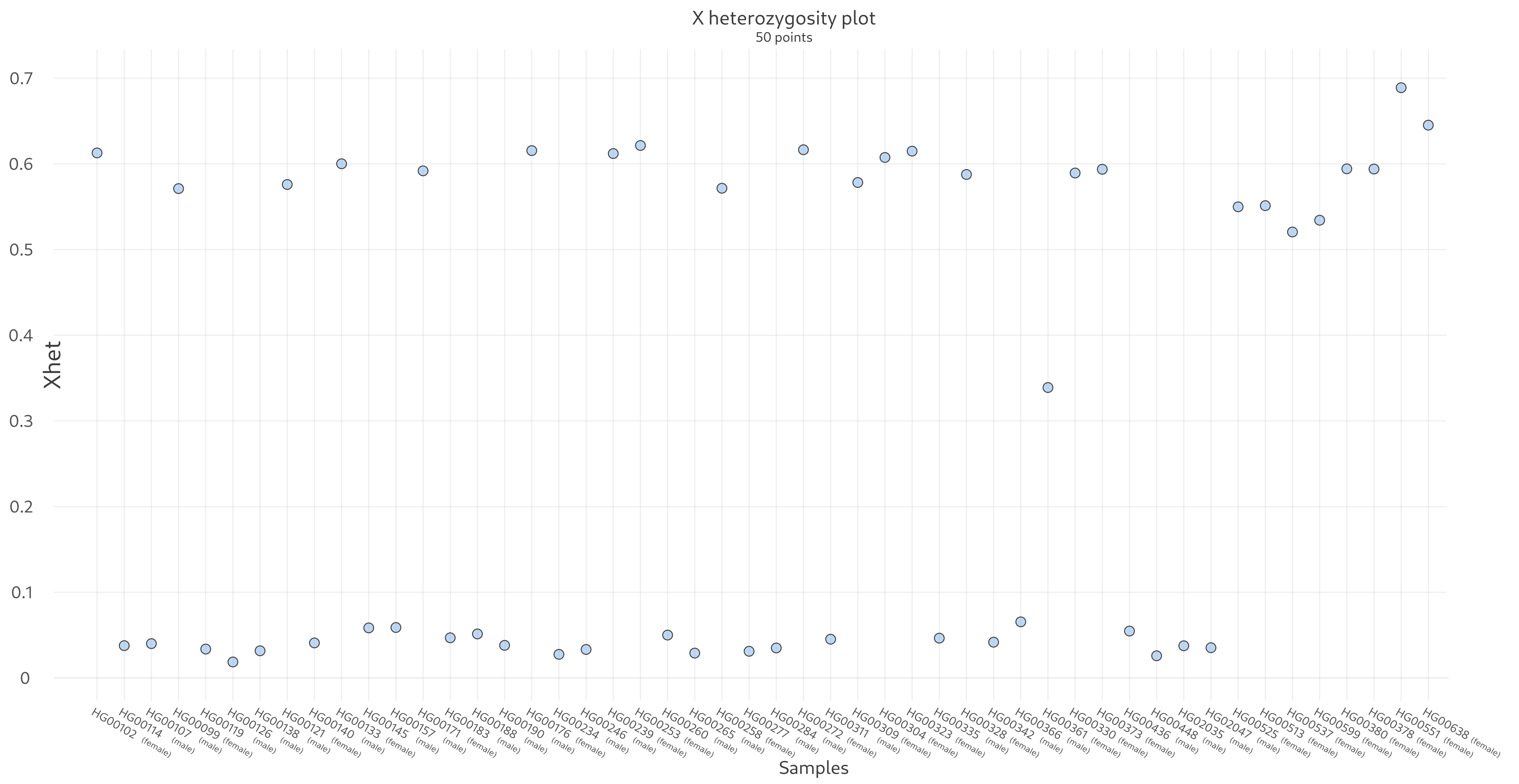
Supplementary Figure 4: X het metric calculated with XhetRel for 50 samples from 1000 genomes project.

## Nextflow pipeline

Nextflow pipeline can be accessed at <https://github.com/barslmn/XhetRel/>. The pipeline automatically pulls the required container for the analysis so only required installations are for nextflow, docker and git. In order to run the pipeline with docker three parameters are required, input and output directory and profile setting to docker.

git clone <https://github.com/barslmn/XhetRel>

nextflow run XhetRel/main.nf --input_dir "/path/to/vcf_files" --output_dir "/path/to/results" –profile docker

**Parameters:**

--input_dir : Directory containing VCF files (required)

--output_dir : Directory for results (required)

-profile docker : Use Docker containers (recommended)

-resume : Resume a previous run from the last successful step

If docker is not available packages can be install manually.

Required tools are MultiQC>=v1.27, vcftools>= v0.1.16, bcftools>=1.20. Example installation for a debian based system:

# Install MultiQC

pip install git+https://github.com/MultiQC/MultiQC

# Install other required tools

apt update -y

apt install -y vcftools

# Install bcftools

git clone --branch 1.20 --recurse-submodules https://github.com/samtools/htslib.git

git clone --branch 1.20 https://github.com/samtools/bcftools

cd bcftools

autoheader && autoconf && ./configure --enable-libgsl --enable-perl-filters

make && make install

cd ..

rm -rf bcftools htslib

## Data availability

All the source code for statistical analysis and visualization can be accessed at <https://github.com/barslmn/XhetRel/blob/master/docs/logisticregression.ipynb>.

## Data for GIAB trios

Data for two GIAB trios has been downloaded from the following paths:

**Chinese trio**

C son:

<https://ftp-trace.ncbi.nlm.nih.gov/ReferenceSamples/giab/data/ChineseTrio/HG005_NA24631_son/HG005_NA24631_son_HiSeq_300x/basespace_45x_bams_vcfs_PerFlowCell/150420_HG005_Homogeneity_01_Combined-23772816/150420-HG005-Homogeneity-01-Combined_S1.vcf>

C father:

<https://ftp-trace.ncbi.nlm.nih.gov/ReferenceSamples/giab/data/ChineseTrio/HG006_NA24694-huCA017E_father/NA24694_Father_HiSeq100x/NA24694_Father_HiSeq100x_fastqs/NA24694_Justin-18411395/NA24694_combined-19068141/NA24694-combined_S1.vcf>

C mother:

<https://ftp-trace.ncbi.nlm.nih.gov/ReferenceSamples/giab/data/ChineseTrio/HG007_NA24695-hu38168_mother/NA24695_Mother_HiSeq100x/NA24695_Mother_HiSeq100x_fastqs/NA24695_Justin-18377364/NA24695_combined-19072106/NA24695-combined_S1.vcf>

**Ashkenazim trio**

AJ son:

<https://ftp-trace.ncbi.nlm.nih.gov/ReferenceSamples/giab/data/AshkenazimTrio/HG002_NA24385_son/NIST_HiSeq_HG002_Homogeneity-10953946/HG002Run02-11611685/HG002-Run2_S1.vcf>

AJ father:

<https://ftp-trace.ncbi.nlm.nih.gov/ReferenceSamples/giab/data/AshkenazimTrio/HG003_NA24149_father/NIST_HiSeq_HG003_Homogeneity-12389378/HG003Run03-13288282/HG003Run03_S1.vcf>

AJ mother:

<https://ftp-trace.ncbi.nlm.nih.gov/ReferenceSamples/giab/data/AshkenazimTrio/HG004_NA24143_mother/NIST_HiSeq_HG004_Homogeneity-14572558/HG004run02-15332344/HG004run02_S1.vcf>
